# Safety culture and adverse event reporting in Ghanaian healthcare facilities: Implications for patient safety

**DOI:** 10.1371/journal.pone.0275606

**Published:** 2022-10-19

**Authors:** Aaron Asibi Abuosi, Collins Atta Poku, Priscilla Y. A. Attafuah, Emmanuel Anongeba Anaba, Patience Aseweh Abor, Adelaide Setordji, Edward Nketiah-Amponsah

**Affiliations:** 1 Department of Public Administration and Health Services Management, University of Ghana Business School, Legon, Ghana; 2 Department of Nursing, College of Health Sciences, Kwame Nkrumah University of Science and Technology, Kumasi, Ghana; 3 Department of Research, Education, and Administration, School of Nursing and Midwifery, University of Ghana, Legon, Ghana; 4 Department of Community Health Nursing, School of Nursing and Midwifery, University of Ghana, Legon, Ghana; 5 Department of Population, Family and Reproductive Health, School of Public Health, University of Ghana, Legon, Ghana; 6 Department of Economics, University of Ghana, Legon, Ghana; Ribeirao Preto College of Nursing at University of Sao Paolo, BRAZIL

## Abstract

**Introduction:**

Recognizing the values and norms significant to healthcare organizations (Safety Culture) are the prerequisites for safety and quality care. Understanding the safety culture is essential for improving undesirable workforce attitudes and behaviours such as lack of adverse event reporting. The study assessed the frequency of adverse event reporting, the patient safety culture determinants of the adverse event reporting, and the implications for Ghanaian healthcare facilities.

**Methods:**

The study employed a multi-centre cross-sectional survey on 1651 health professionals in 13 healthcare facilities in Ghana using the Survey on Patient Safety (SOPS) Culture, Hospital Survey questionnaire. Analyses included descriptive, Spearman Rho correlation, one-way ANOVA, and a Binary logistic regression model.

**Results:**

The majority of health professionals had at least reported adverse events in the past 12 months across all 13 healthcare facilities. Teamwork (Mean: 4.18, *SD*: 0.566) and response to errors (Mean: 3.40, SD: 0.742) were the satisfactory patient safety culture. The patient safety culture dimensions were statistically significant (χ2 _(9, N = 1642)_ = 69.28, *p* < .001) in distinguishing between participants who frequently reported adverse events and otherwise.

**Conclusion:**

Promoting an effective patient safety culture is the ultimate way to overcome the challenges of adverse event reporting, and this can effectively be dealt with by developing policies to regulate the incidence and reporting of adverse events. The quality of healthcare and patient safety can also be enhanced when healthcare managers dedicate adequate support and resources to ensure teamwork, effective communication, and blame-free culture.

## Introduction

In healthcare, adverse events are an ever-occurring reality. Safety has therefore become the heart of healthcare quality [1]. Efforts are needed to strengthen healthcare systems and enhance a positive attitude of the workforce toward patient safety culture. With every health organization, values explain what is significant in the system, and it includes the expectation from health staff, their attitudes and behaviour, and processes on reward or otherwise. These important parameters are paramount to achieving patient safety. It is therefore critical to focus on system improvement to increase patient safety [1, 2].

Safety culture also referred to as safety climate in any organization is determined by the health and safety management philosophy and efficiency. It represents the outcome of group and individual beliefs, attitudes, values, and behaviour in ensuring safety in an organization. Typically, the idea of patient safety culture is classified into different dimensions such as leadership, workload, communication, collaboration or teamwork, effective safety systems, non-punitive response to errors, and commitment to safety by management [3, 4]. Recognizing the values and norms that are significant to healthcare organizations are the prerequisites for establishing a patient safety culture. Understanding the safety culture is essential for improving undesirable workforce attitudes and behaviours including miscommunication on adverse incidents; thereby instituting non-punitive approaches to errors by managers, which can improve the safety culture in healthcare systems. Similarly, an effective assessment of the safety culture can aid in identifying problem areas that could lead to errors and unfavourable outcomes [5]. Because the health team is composed of different professionals and units that make up a bigger healthcare organization, the influence of the group’s values, beliefs, and actions can have a greater impact than the function of a single professional group. The significance of developing a safety culture has been emphasized since failures in healthcare organizations have been linked to weak adverse events reporting principles [6].

Adverse events as defined by the World Health Organization (WHO) are errors or mistakes in healthcare that lead to demonstrable harm or injuries to patients and are unrelated to the underlying condition [7, 8]. It includes equipment and medication errors, inefficiencies in making a clinical decision, hospital-acquired infection, misdiagnosis, device loss [8], pressure ulcers, post-operative complications, increased complaints from patients and relatives [9], and other challenges that risk quality care delivery [10].

In a WHO report on the adverse event in African and Eastern Mediterranean, medical errors accounted for 34%, diagnostic errors (19%), obstetrical errors (18%), and neonatal harm (9%) with 20% of patients being harmed; with a third of all cases resulting in mortalities [11]. The majority of adverse events brought on by medical errors happen in low- and middle-income countries (LMICs). About a tenth of the 421 million hospitalizations annually around the world are considered to be at risk, with LMICs accounting for about two-thirds of all occurrences [8, 12].

This incidence as linked with healthcare is alarming, with the staggering annual cost of detectable medical errors, as it is projected to result in financial losses equivalent to billions of dollars based on data from previous years in the US alone [13]. The highest annual costs were incurred by the two most frequent classes of adverse events, post-surgical infections and bed sores (6.5 billion USD). Besides the listed events, infections related to infusions, injections, and other procedures cost the healthcare system an additional cost of over one billion USD [10, 14].

Consequently, millions of the affected patients are complicated to impairments, disabilities, or even fatalities [15]. Adverse events impact patient safety and healthcare quality, and it is noted to also account for the poor quality of life and significant mortalities of patients from re-hospitalization [16–19]. Moreover, in the event of poor patient safety culture in healthcare, an increased incidence of the adverse event has negatively affected the morale and competence of healthcare workers, and this has significantly impacted health outcomes [20, 21]. Against the background of relatively increased rates of avertible patient safety issues, the health system’s focus has shifted to enhancing patient care quality through improving patient safety culture [22, 23].

Globally, the distribution of adverse events remains a pertinent concern. Iatrogenic causes of adverse events are noted to contribute greatly to clinical mortality, and this calls for the attention of all stakeholders [9, 24, 25]. Nonetheless, most of the healthcare blunders that result in adverse events are not caused by the lack of competence or skills of the healthcare personnel. More often than not, adverse events stem from issues with healthcare provision caused by factors at the level of the patient, the clinical procedure, or the health workforce. Adverse events could even be rooted in the practice environment [23], with examples of unsafe physical structures, dysfunctional equipment, unhealthy workload, treatment delays, and even misdiagnosed conditions [26, 27].

Reason [25] asserts that adverse events occur if defences along the causal chain fail. It is important to accept that some adverse events are unavoidable; as it extends from the product of systemic breakdowns during procurement of the material resources through its management. Blaming health personnel or engaging in punitive behaviour to avert adverse events is not effective in addressing patient safety issues [28, 29]. It is therefore essential for the health system to accept that adverse events are not always the consequence of incompetence and can occur without the provider’s negligence [26].

Importantly, timely and reliable reporting of adverse events is critical for organizational learning and the improvement of more dependable healthcare systems [10, 30, 31]. According to Hessels et al. [32], the safety culture in healthcare organizations impacts the rate of adverse event reporting. Patient safety culture, particularly in hospitals, is built on mutual trust, appropriate communication, organizational learning, shared perceptions of the essence of safety, healthcare leadership commitments, and the existence of a non-punitive plan for dealing with the incidence of adverse events reporting [18, 33].

Globally, the rates of adverse event reporting range from 10 to 80 per cent [8], with significant rates in high-income countries (HICs) such as Canada [34], China [35, 36], the United Kingdom [32] and the United States [37] and LMICs such as Iran [38] and East Africa [39]. Strategies for the prevention of harm and other errors in healthcare settings require an understanding of the different types of adverse events and their prevalence [40]. Regrettably, few studies have been done on the nature of patient safety culture in LMICs, given the substantial harm caused by adverse events. The majority of the health workforce in LMICs lack knowledge and a clear understanding of the patient safety culture status in their healthcare facilities. This is mostly due to the absence of a conventional tool for reporting such incidents. Notwithstanding the lack of synchronised nationwide reporting systems in most developing countries, adverse events reporting by healthcare practitioners at the time of care is still the more recognized and effective method for identifying genuine errors and near misses in care delivery [35, 38, 39]. Adverse event reporting promotes organizational learning and improves the pace at which improvements might be made. In magnet facilities, effective reporting does not just address adverse events but other common safety issues are effectively taken care of because it is gathered from various settings [5].

In sub-Saharan Africa (SSA), information about the importance and influence of patient safety culture on quality care delivery is inadequate and therefore needs thorough research on the status of healthcare facilities [41]. Even though various studies have found a link between the workforce’s perceptions of patient safety culture and the frequency of adverse events reported globally, there is inadequate data in most LMICs including Ghana. Most reported events are associated with adverse events following immunization (AEFI), which is even considered low with reporting ratio of 1.56 per 100,000 infants despite the huge public health attention given to it [42]. Determining the rate of adverse event reporting and the factors that accounts for it is key for preventive strategies [38]. It is critical to boost the documentation of the frequency of adverse events and communicate appropriate information about the nature and possible prevention of unfavourable adverse events among healthcare communities [26]. This can only be achieved in LMIC settings, which are without a properly developed incident reporting system through synthesising adequate data through research on patient safety culture and adverse event reporting. The study, therefore, assessed the frequency of adverse event reporting, the patient safety culture determinants of the frequency of adverse event reporting and the implications for Ghanaian healthcare facilities.

## Materials and methods

### Study design and setting

This study employed a multi-centre cross-sectional survey conducted among health professionals in thirteen (13) healthcare facilities in Ghana. Ghana is made up of 16 administrative regions in three ecological zones. Three regions including the Bono, Greater Accra, and Upper East regions were randomly selected, each from the southern, middle, and northern ecological zones. The total health workforce in Ghana stands at 122,183 in 1044 healthcare facilities. The healthcare facilities are grouped into hospitals (teaching, regional, municipal/district), health centres, clinics, and community-based health planning and services (CHPs) compounds. The Greater Accra region has 438 health facilities and the proportionate highest number of health professionals, 120 health facilities in the Bono region, and 211 health facilities in the Upper East region [43, 44]. A total of 13 healthcare facilities were purposely selected for the study. This comprised four healthcare facilities each from the Bono and the Upper West regions (district, faith-based, private, and regional hospital). Five facilities comprising teaching, regional, district, faith-based, and private hospitals were also purposively selected from the Greater Accra region for the study. The choice of the facilities was informed by the diversity of the working environment across the various levels of care. A teaching hospital was also selected due to the availability of specialised services.

### Participants

The participants were health workforce in the selected healthcare facilities. The population included both clinical and non-clinical staff comprising nurses, doctors, pharmacists, laboratory technologists and technicians, and administrative and support staff. Healthcare professionals (N  = 1651), including registered nurses and midwives (n  =  976), physicians (n  =  132), pharmacist and pharmacy technicians (n = 47), laboratory technologists and technicians (n = 45), dieticians (n = 11), nursing and healthcare assistants (n  =  264), other clinical staff (n = 9) and non-clinical staff (n = 167) participated in the study. Full-time health workers with more than a year of working experience and accepted to be part of the study were included in the study. Healthcare professionals on leave were excluded from the study.

### Sample and sampling technique

A multistage sampling approach was adopted; a proportionate stratified sampling was used to allocate the sample for each region and facility; however, the convenience sampling method was used to select the participants from the study sites, as it was appropriate for the study. A total of 2000 questionnaires were distributed with a response rate of 1701 (86.2%), however, 1651 of the questionnaires were accurately completed.

### Measures

#### Socio-demographic characteristics

The participant’s socio-demographic characteristics, including age, sex, educational level, marital status, region, primary work area, position, hours per week, and work experience were measured.

#### Patient safety culture

The Survey on Patient Safety (SOPS) Culture, Hospital Survey questionnaire (version 2.0) was adapted from the Agency for Health Research and Quality for data collection [45]. The questionnaire has 32 items categorized into 10 dimensions, including teamwork (3 items); staffing and work pace (4 items); organizational learning—continuous improvement (3 items); response to error (4 items); supervisor, manager, or clinical leader support for patient safety (3 items); communication about the error (3 items); communication openness (4 items); reporting patient safety events (3 items); hospital management support for patient safety (3 items); handoffs and information exchange(3 items). All the items were on a Likert scale ranging from (1 = strongly disagree/never; 2 = disagree/rarely; 3 = neither agree nor disagree/sometimes; 4 = agree/ most of the time; and 5 = strongly agree/ always). The mean score of the items in each dimension was computed to a total score and divided by the number of items in the dimension. The scale has an acceptable reliability score (Cronbach alpha) ranging from 0.63 to .84, and also reported a good discriminant and convergent validity [46–48].

#### Frequency of adverse event reporting

The frequency of adverse event reporting was measured using the reporting patient safety event dimension from the Survey on Patient Safety (SOPS) Culture, Hospital Survey questionnaire (version 2.0). The dimension has three (3) items; however, a single item was used to assess the frequency of reporting adverse events in the past 12 months. It was based on the frequency of “none”, “1 to 2”, “3 to 5”, “6 to 10” and “11 or more” [45–47].

#### Data collection

Data collection started in July 2021 and ended in September 2021. After securing permission from the management of the various study sites, prospective healthcare professionals were informed about the study. The questionnaires were administered to the participants by the researchers after they consented to the study during morning and after shifts. The researchers collected the participants and completed the questionnaires.

#### Data analysis

Data analysed was done with the use of SPSS (Version 26.0). Descriptive statistic was used in the analysis of the socio-demographic characteristics, frequency of the adverse event reported, and patient safety culture using frequencies, percentages, mean and standard deviations. A one-way analysis of variance (ANOVA) was conducted to compare the frequency of adverse event reporting among the 3 levels of care. The response for adverse event reporting was also dichotomised into “reporting” or “not reporting”. A binary logistic regression analysis model was used to determine the relationship between patient safety culture variables as independent variables and the adverse event reporting as the dependent variable, after a Spearman Rho Correlation analysis was conducted. The variance inflation factor was checked, and no multicollinearity among the explanatory variables was seen. The test also ensured that the assumption of homogeneity of variance was not violated. The analysis was conducted at a 95% confidence level.

#### Ethical consideration

Letters of the request were sent to the various management units of the hospitals to seek permission to use the facilities. Ethical approvals for the study were sought from two Institutional Review Boards; the Ghana Health Service Ethics Review Committee (GHS-ERC: 007/04/21) and the Ethics Committee for the Humanities, University of Ghana (ECH 109/ 20–21). In addition, written informed consent was sought from all participants before data were collected. It was also explained to participants that participation in this study was voluntary. Anonymity and confidentially were ensured by using not requesting the names of participants.

## Results

### Socio-demographic characteristics of participants

The mean age of the participants was 33.6(±6.38) years. More than half of the participants were female (*n* = 912; 55.2%), married (*n* = 1080; 65.4%), and worked in the primary level of care (*n* = 766; 46.4%). Further, most of the participants were nursing staff (n = 1197, 72.5) and worked in the medical-surgical unit (*n* = 678, 41.1%) and had worked for at most 5 years in the present healthcare facility (*n* = 1127; 68.3%). Additionally, most of the participants work for an average of 30 to 40 hours per week (n = 910, 55.1%). Table 1 presents the detailed socio-demographic data of participants and the hospital characteristics.

**Table 1:** Socio-demographic data of participants and hospital characteristics.

Demographic Data	Mean	SD
**Age**	33.60	6.38
**Sex**	**N**	**%**
Male	739	44.8
Female	912	55.2
**Marital status**		
Married	1080	65.4
Never married /Widowed/Divorced	571	34.6
**Level of care**		
Primary	766	46.4
Secondary	450	27.3
Tertiary	435	26.3
**Primary Unit**		
Medical-Surgical Units	678	41.1
Emergency care	108	6.5
Obstetrics and Gynaecology Unit	292	17.7
Paediatric Unit	141	8.5
Specialist Service Units	184	11.1
Diagnostic and Other Clinical Services	128	7.7
Administrative and Support Services	120	7.3
**Position in hospital**		
Nursing Staff	1197	72.5
Medical Officer	175	10.6
Other Clinicians	141	8.5
Managerial, Admin. and Support Staff	138	8.4
**Number of years in facility**		
Less than 2years	286	17.3
2 to 5 years	841	50.9
More than 5 years	524	31.7
**Number of hours per week**		
30 to 40 hours per week	910	55.1
More than 40 hours per week	741	44.9

### Patient safety culture and frequency of adverse events

Out of 1,651 participants, the vast majority (n = 1149, 69.6%) had at least reported adverse events in the past 12 months. While the mean score of the patient safety grade was 2.33 (*SD*: 0.622), the mean score of patient safety culture was 3.75 (SD: 0.652) with the Teamwork Subscale (mean: 4.18, *SD*: 0.56) and response to errors (mean: 3.40, SD: 0.74) revealed as the highest and lowest rated subscales respectively. Table 2 provides the detailed frequency, per cent, mean, and standard deviations of adverse event reporting, patient safety culture, and safety grade of the healthcare facilities.

**Table 2:** Descriptive statistics of adverse events frequency and patient safety culture.

Variable	Frequency (N)	Per cent (%)	Mean (SD)
**Frequency of Adverse Event**			
None	502	30.4	
1 to 2	511	31.0	
3 to 5	496	30.0	
6 or above	142	8.6	
**Patient Safety Culture Dimensions**			
Teamwork			4.18 (.566)
Organizational learning			3.90 (.623)
Response to error			3.40 (.742)
Supervisor support for PS			3.94 (.587)
Communication about error			3.96 (.627)
Communication openness			3.84 (.667)
Hospital management support PS			3.52 (.757)
Handoffs and information exchange			3.88 (.671)
Staffing			3.41 (.625)
Total patient safety culture			3.78 (.652)
**Patient Safety Grade**			2.33 (.622)

### Comparison of adverse event reporting and patient safety perception among the levels of care

A one-way ANOVA was conducted to understand if there are differences in the frequency of adverse events reporting and perception of patient safety culture on the three levels of care. Participants were grouped according to their level of care (tertiary, secondary, and primary). Table 3 displays a statistically significant difference at the *p* < .05 level in the frequency of adverse event reporting for the three levels of care: *F*_(2, 1648)_ = 25.678, *p <* .01; the difference in mean scores between the groups was however small. The effect size, calculated using eta squared, was 0.03. The difference in the perception of patient safety on the levels of care, was also statistically significant (*F*_(2, 1648)_ = 8.872, *p <* .01). As shown in Table 4, post-hoc comparisons using the Tukey HSD test indicated that the mean score on adverse event reporting for tertiary facilities (*M* = 1.44, *SD* = .917) was significantly different from secondary facilities (*M* = 1.12, *SD* = .949) and primary facilities (*M* = 1.04.96, *SD* = .959). Secondary facilities (*M* = 22.10, *SD* = 4.15) statistically did not differ significantly from primary facilities in the frequency of adverse event reporting. Similarly, while there were differences in perception of patient safety in tertiary level of care and primary (*M* = .45, *SD* = .947) and secondary (*M* = .43, *SD* = .496), no statistically significant difference was reported between primary and secondary care facilities on the perception of patient safety.

**Table 3:** One-way ANOVA of the frequency of reporting adverse events on three levels of care.

	Tertiary (n = 435)			Secondary (n = 450)			Primary (n = 766)		
Variables	M	SD	SE	M	SD	SE	M	SD	SE
Frequency of events reported	1.44	.917	.044	1.12	.949	.045	1.04	.959	.035
Perception of patient safety culture	.33	.469	.023	.43	.496	.023	.45	.497	.018

**Table 4:** Post-hoc Tukey HSD test.

Dependent Variable	Level of care (I)	Level of care (J)	MD (I-J)	SE	Sig.
Frequency of events reported	Tertiary	Secondary	.319*	.064	.000
		Primary	**.401** *	.057	.000
	Secondary	Tertiary	-.319*	.064	.000
		Primary	.082	.056	.313
	Primary	Tertiary	**-.401** *	.057	.000
		Secondary	-.082	.056	.313
Patient safety perception	Tertiary	Secondary	-.105*	.033	.004
		Primary	-.120*	.029	.000
	Secondary	Tertiary	.105*	.033	.004
		Primary	-.015	.029	.858
	Primary	Tertiary	.120*	.029	.000
		Secondary	.015	.029	.858

*. The mean difference is significant at the 0.05 level. MD–mean difference

### The relationship between the frequency of adverse event reporting and patient safety culture

The frequency of adverse event reporting and the dimensions of patient safety culture was examined using the *Spearman Rho* correlation coefficient as presented in Table 5. There was a weak, positive correlation between the frequency of adverse event reporting and patient safety culture variables as: teamwork (*r* = .163, *p* < .01), organizational learning (*r* = .132, *p* < .01), response to error (*r* = .063, *p* < .05), supervisor support for patient safety (*r* = .139, *p* < .01), hospital management support patient safety (*r* = .081, *p* < .01), and staffing (*r* = .142, *p* < .01).

**Table 5:** Correlations between frequency of adverse events reported and patient safety culture.

	1	2	3	4	5	6	7	8	9	10
**1.** Frequency of Adverse Events Report	1									
**2.** Teamwork among staff	.163**	1								
**3.** Organizational learning	.132**	.453**	1							
**4.** Response to error	.063*	.390**	.412**	1						
**5.** Supervisor support for PS	.139**	.431**	.492**	.434**	1					
**6.** Communication about error	.009	.212**	.276**	.251**	.253**	1				
**7.** Communication openness	.045	.242**	.271**	.426**	.344**	.467**	1			
**8.** Hospital management support on PS	.081**	.106**	.247**	.197**	.220**	.258**	.210**	1		
**9.** Handoffs and information exchange	.009	.207**	.305**	.283**	.309**	.107**	.184**	.218**	1	
**10.** Staffing	.142**	.338**	.429**	.366**	.355**	.117**	.168**	.108**	.240**	1

**Correlation is significant at the 0.01 level (2-tailed)

*Correlation is significant at the 0.05 level (2-tailed)

### Safety culture predictors of frequency of adverse event reporting

Binary logistic regression was performed to assess the prediction of the patient safety culture dimensions on the frequency of adverse event reporting in healthcare settings. The model contained nine independent variables. The full model was statistically significant, χ2 _(9, N = 1642)_ = 69.28, *p* < .001, indicating that the model was able to distinguish between participants who frequently reported adverse events and those who did not report them. The model explained between 5.0% (Cox and Snell R square) and 7.4% (Nagelkerke R squared) of the variance in frequency of adverse event reporting and classified 66.0% of cases. In Table 6, the details of the seven predicting variables (Teamwork, response to error, supervisor support for patient safety, communication about error, hospital management support for patient safety, handoffs and information exchange, and staffing) contributed statistically to the model has been reported. The binary logistic regression analysis indicates that the chance of reporting adverse events is 1.43 higher among those who reported (compared to those who did not report) [95% CI: 1.129–1.804] for each communication about errors in the healthcare settings by the workforce. Similarly, the likelihood of reporting adverse events in the healthcare setting is 0.56 higher among those who reported adverse events when there is teamwork among staff [95% CI -.421-.749] when all other factors are controlled in the model.

**Table 6:** Logistic regression predicting adverse event reporting in the healthcare setting.

	B	S.E.	Wald	Df	Sig.	Odds Ratio	95% CI for OR	
							Lower	Upper
Teamwork	-.577	.147	15.457	1	**.000**	.56	.421	.749
Organizational learning	-.143	.133	1.147	1	.284	.87	.668	1.126
Response to error	.226	.106	4.528	1	**.033**	1.25	1.018	1.545
Supervisor support for PS	-.435	.148	8.628	1	**.003**	.65	.484	.865
Communication about error	.356	.120	8.853	1	**.003**	1.43	1.129	1.804
Communication openness	-.046	.118	.153	1	.696	.96	.757	1.204
Management support of PS	-.126	.092	1.872	1	.171	.89	.735	1.056
Handoffs and info exchange	.242	.104	5.438	1	**.020**	1.27	1.039	1.562
Staffing	-.252	.120	4.409	1	**.036**	.78	.614	.983
Constant	4.181	.726	33.133	1	.000	65.44		

a. Variable(s) entered: Teamwork, Organizational learning, Response to error, Supervisor support for patient safety, Communication about the error, Communication openness, Hospital management support of patient safety, Handoffs and information exchange, Staffing.

## Discussion

Adverse event reporting is regarded as a significant indicator in assessing patient safety culture in the healthcare system; as it serves as an indispensable tool in promoting safety and quality monitoring. The health workforce is instrumental in achieving such a culture [49]. The results of the current study add to the expanding knowledge on patient safety culture by describing the prevalence of adverse events in healthcare and emphasizing the value and the role of healthcare managers, teamwork, and organizational support in adverse event reporting [9, 32].

The findings of the study are congruent with the outcome as reported by Schwendimann et al [8] and Hibbert et al [50] as the most acknowledged adverse event in the form of medication errors, falls, surgical sites infections and bed sores are prevalent in healthcare settings. Though according to de Vos et al [51], whether there are adverse events or not during admissions, the majority of patients register complaints such as falls, medication errors, pressure sores, etc as typically about challenges of quality and safety problems in the healthcare environment. It has been determined that adverse event occurrences account for 55.6% of complaints made during admissions, or around one out of every three [51]. Adverse events which are identified by healthcare professionals through observation and complaints from patients and/or relations are a particularly underutilized source of information for improvement in most care settings because they frequently remain completely isolated from quality and safety data. The current study on the patient safety culture in healthcare settings offers a hint as to the potentially overlooked incidence of safety that all stakeholders should be concerned about, and healthcare managers should be dynamic in using all applicable tools to assess information on adverse events.

As a means of improving the patient safety culture, frequency and accuracy of reporting adverse events in healthcare facilities are necessary. The study assessed the patient safety culture determinants on the frequency of adverse event reporting. It is imperative to note that organizations with electronic health record systems potentially have easier means of reporting adverse events [9, 19]. Best practices in the field of patient safety on adverse event reporting, consistent with the current study findings have been reported in the United States [32], China [30, 52], the Philippines [49], and Norway [53]. With such an encouraging higher reporting rate of adverse events in the past year, the health workforce must be motivated to improve the frequency of their reporting and communication, especially with healthcare managers. Alternatively, it is recommended for healthcare organizations to establish electronic media for easy and quick monitoring and reporting the adverse events. The episodes of poor reporting format for adverse events and barriers including consequences associated with blame or unjust culture, procedural and managerial gaps can be addressed to encourage the workforce to report all forms of adverse events in the facilities [54, 55].

The satisfactory patient safety culture in the Ghanaian healthcare facilities was consistent with studies in other LMICs such as Iran [56, 57], Ethiopia [58], and Jordan [40] though Kakemam et al’s [33] report were incongruent with the current study. The variations among HICs and some LMICs may be due to organizational commitment to patient safety issues in the respective countries concerning management support, teamwork, and resources to address patient safety challenges. The patient safety culture will need continuous improvement as its attainment is a process, not an event. Policymakers must therefore pay attention on the successes in the healthcare industry by maintaining and strengthening the various component of the patient safety culture. Since globally, the concept of patient safety culture is quite a new concept, healthcare managers must be open and welcoming to future challenges that may interrupt full integration in the Ghanaian healthcare system.

Though successes were reported, patient safety was graded below average. This is in contrast to findings from hospitals in China [59], South Korea [60], and Europe [61]. The perception might have resulted from poor staff skill mix and its associated quality of care, and inadequate reporting and documentation nationally on patient safety. Healthcare leadership keeping pace with international practice on adverse events and other patient safety communication will go a long way to improving the safety of the patient in health facilities.

Globally, the health workforce is required to report adverse events, and most agree that this obligation increases patient safety. Although the perception and attitude of the health team towards reporting adverse events is crucial for patient safety, Moinuddin et al found that staff in tertiary facilities have little awareness and negative perception and usually do not report adverse events [62]. This development is in contrast to the current finding which found a comparatively higher reporting rate in tertiary facilities. The reason may be due to the complex nature of care in the various units in tertiary facilities including intensive care units, operating theatres, and emergency departments with its increased rate of adverse events [63]. The finding is supported by similar findings which indicated that the larger facilities have a more advanced reporting system that possibly improves the reporting practice [64]. Though the underlying determinants of underreporting are many; the need for patient safety training, commensurate workload, and reduced blame culture will improve from adverse event reporting to facilitate learning and quality improvement.

Largely, there is a relationship between dimensions of the patient safety culture and the frequency of adverse event reporting. Though most patients refuse to report the harm they suffer, most health personnel also overlook an environment where they are likely to be blamed unreasonably. The outcome of the current study is consistent with other findings that reported a correlation between patient safety culture and frequency of adverse event reporting [54, 56, 65].

The dimensions of patient safety culture predict the frequency of adverse events reported. In a practice environment with positive safety culture manifested by teamwork, non-punitive response to error, manager support, adequate staffing and handoffs, communication about errors, and information exchange, there is an increased chance of adverse events reported. Hessels et al. [32] assert that adequate response to adverse events and open communication about an error in the practice environment and hospital management’s support for patient safety improves adverse event reporting. In most settings, adverse events are missed during admissions only to resurface as readmissions, and in extreme cases as mortalities [57, 66].

Kakemam et al. [33] report that constant effort through teamwork is essential in promoting adverse event reporting. Mutual support between the workforce and strong teamwork increases the likelihood of adverse event reporting in healthcare settings [67]. Healthcare leadership is fundamental for promoting adverse event reporting in the healthcare setting. Open discussions and implementation of training programmes on patient safety reduce adverse event reporting. Leaders are therefore advised to advance strategies that promote an effective system that reduce the phenomenon of blaming the workforce that reports errors [68].

As patient safety culture is considered a pivotal indicator of healthcare, the current study affirms the possible role of communication between healthcare providers about errors. In putting up an action plan to address and/or improve the phenomenon, communication between the health workforce and the rest of the healthcare team, especially the patient. This position as asserted by Gallagher and Mazor [69] and Skär and Söderberg [70] will boost the active participation of patients in their care and enhance confidence among the health workforce. Similarly, the findings of the current study agree with Jorro Barón et al [71] and Shahid and Thomas [72] on the role of hand- and taking-over of patients in adverse event reporting. Continuity of care in the healthcare system is achieved when there is an adequate exchange of clinical information between healthcare providers; however, communication gaps in healthcare delivery could lead to an increased rate of medical errors. Healthcare leaders are encouraged to standardize an effective communication approach within the clinical space in the provision of safe care.

It is established that inadequate staffing is associated with burnout and poor patient safety. Holistic determination of the staffing levels by healthcare managers will go a long way to address the issue that can potentially affect the issue of patient safety especially adverse event reporting [27, 73]. The findings of this study indicated that the level of staffing could predict adverse event reporting; the healthcare system can therefore be improved in the milieu of both material and human resources [74]. However, the dynamics of improved adverse event reporting relative to safe staffing cannot be oversimplified, as other elements such as leadership [75], teamwork [76], work environment [77], and communication [70] come into play. Adequate healthcare financing to address human and material resources against the background of complexities of growing global demand for health workforce amid COVID-19 can address the challenges associated with patient safety [78, 79].

### Limitations

Though few studies have been conducted on adverse event reporting in sub-Saharan Africa, this study is considered a novel work in Ghana relative to the influence of patient safety culture. There were, however, identified limitations to the study findings. The data were collected during the COVID-19 pandemic, which could potentially affect the outcome of the study. Due to the cross-sectional approach of the study, the researcher cannot establish a causal effect of patient safety culture on adverse event reporting. Additionally, the data on patient safety variables was self-reported by the healthcare providers; more rigorous data collection techniques such as participant observation could have yielded more reliable data. However, the choice of a validated tool and adequate sample size for the study addressed the challenge.

Future studies could consider employing a mixed-method approach that will afford healthcare providers the chance to share their experience with the phenomenon. Studies that highlight the role of electronic record systems on effective adverse event reporting should be considered. Additionally, the impacts of burnout, job demand, and resource on adverse event reporting and patient satisfaction can be researched to provide clarity on the other factors that account for the phenomenon.

### Implications for healthcare management

Leadership’s role in overcoming the challenges of adverse events reporting can effectively be dealt with by developing a practice environment policy to regulate the incidence and reporting of the events. Additionally, healthcare managers should invest in high ethical standards within the workplace, which centre on open communication and blame-free culture. Such initiative will drive the workforce to openly and transparently report errors associated with healthcare. Meanwhile, the episodes of adverse events can equally be reduced through improved teamwork among the healthcare providers, and human and material resources with a realistic workload [80, 81].

Also, a training programme designed to address the caring behaviour of healthcare providers can be initiated to serve as a catalyst to address adverse event reporting. Committing to the value of care can be improved if leadership facilitates an environment of judicious documentation and communication of healthcare activities [49]. Promoting empowerment and open dialogue between the healthcare staff will promote a practice environment that advances patient safety [82].

Moreover, efforts to lessen the obstacles to adverse event reporting can wholly improve patient safety. Barriers reported in past studies include inadequate knowledge of the main incidents and the procedure for reporting them. Introducing in-service training on adverse events by healthcare managers can go a long way in enhancing the effective reporting culture. Again, data support the idea that the right attitudes toward adverse event reporting begin to develop during the training of health personnel, it is therefore recommended that adverse events should be made an integral component of the curriculum of the health workforce [83].

A major setback as identified in adverse event reporting is the system of unjust culture of penalising staff involved with adverse events. Healthcare managers should advance steps that will assist the workforce to overcome the fear of being scolded or sanctioned when there are adverse events; by developing new and/or improving existing systems that focus on safety and quality development centred on supporting staff in the event of medical errors. Adopting contemporary reporting technologies such as the electronic health record system by the health workforce can ease the challenges of getting data on adverse events, especially in LMICs [84–87].

With the increasing rates of health workforce turnover and the intricacies in the practice environment, developing a well-informed and comprehensive training plan and improving budget allocation to cater for material resources and staffing that are responsive to the healthcare needs of SSA is key.

## Conclusions

In assessing the patient safety culture predictors of adverse event reporting; the findings have contributed to the knowledge of the increasing pool of data on patients’ safety. By inference, we cannot make a conclusive statement on adverse events reported per the data in the current study; the quality of healthcare and patient safety can, however, be enhanced when healthcare managers and policymakers dedicate adequate attention to ensuring teamwork, communication, and staffing.

The ability of the health professional to voluntarily report adverse events can be improved by creating a patient safety culture through teamwork, managers’ responsiveness to errors, open communication about errors, adequate handoffs and information exchange among staff, and adequate staffing. This study supported other research conclusions that patient safety culture predicts adverse event reporting and that a rise in patient safety culture is associated with a reduction in the frequency of adverse events. To enhance the reporting rate, it is advised for hospital managers to put in place a non-punitive reporting system while rewarding staff who report adverse events.
